# Study of Overprotective-Polarization of Steel Subjected to Cathodic Protection in Unsaturated Soil

**DOI:** 10.3390/ma14154123

**Published:** 2021-07-24

**Authors:** Mandlenkosi G. R. Mahlobo, Peter A. Olubambi, Phumlani Mjwana, Marc Jeannin, Philippe Refait

**Affiliations:** 1 Centre for Nanoengineering and Tribocorrosion, University of Johannesburg, Johannesburg 2028, South Africa; mahlobomgr@yahoo.com (M.G.R.M.); polubambi@uj.ac.za (P.A.O.); mjwanap@gmail.com (P.M.); 2 Laboratory of Engineering Sciences for the Environment (LaSIE)—UMR 7356, University of La Rochelle/CNRS, 17000 La Rochelle, France; mjeannin@univ-lr.fr

**Keywords:** cathodic protection, carbon steel, polarization level, EIS, voltammetry, unsaturated soil

## Abstract

Various electrochemical methods were used to understand the behavior of steel buried in unsaturated artificial soil in the presence of cathodic protection (CP) applied at polarization levels corresponding to correct CP or overprotection. Carbon steel coupons were buried for 90 days, and the steel/electrolyte interface was studied at various exposure times. The coupons remained at open circuit potential (OCP) for the first seven days before CP was applied at potentials of −1.0 and −1.2 V vs. Cu/CuSO_4_ for the remaining 83 days. Voltammetry revealed that the corrosion rate decreased from ~330 µm yr^−1^ at OCP to ~7 µm yr^−1^ for an applied potential of −1.0 V vs. Cu/CuSO_4_. CP effectiveness increased with time due to the formation of a protective layer on the steel surface. Raman spectroscopy revealed that this layer mainly consisted of magnetite. EIS confirmed the progressive increase of the protective ability of the magnetite-rich layer. At −1.2 V vs. Cu/CuSO_4_, the residual corrosion rate of steel fluctuated between 8 and 15 µm yr^−1^. EIS indicated that the protective ability of the magnetite-rich layer deteriorated after day 63. As water reduction proved significant at this potential, it is proposed that the released H_2_ bubbles damage the protective layer.

## 1. Introduction

Buried carbon steel is very susceptible to corrosion when exposed to soil environments [1,2,3,4,5,6,7,8,9,10,11,12]. Underground pipelines must then be protected from external corrosion, which is achieved using the combination of organic coatings and cathodic protection (CP). Common coatings used on pipelines, past or present, are high build epoxy, coal tar, polyethylene, polyolefin and polyurethane [13]. Combined with a coating, CP protects the parts of the pipeline surface that are in contact with the soil because the coating is damaged or defective. Standards such as NF EN 12954 and NF EN ISO 15589-1 [14,15] specify the protection potential to be applied to achieve an efficient cathodic protection, i.e., to ensure a “residual” corrosion rate (that is the corrosion rate achieved with CP) lower or equal to 10 µm yr^−1^. According to the NF EN 12954 standard, the potential required for the protection of steel buried in aerated unsaturated soils is, in most cases, −0.85 V vs. Cu/CuSO_4_. At such potential, in aerated conditions, the main cathodic process is the reduction of dissolved oxygen:

(1) $$O_{2}+2H_{2}O+4e^{-}\to 4{\mathrm{OH}}^{-}$$

This potential value is, however, only a threshold and a further decrease of the potential would in principle lead to a further decrease of the residual corrosion rate. For instance, at −1.0 V vs. Cu/CuSO_4_, CP is expected to be more efficient. At lower potentials, the reduction of H_2_O, i.e., the hydrogen evolution reaction (HER), may also take place:

(2) $$2H_{2}O+2e^{-}\to H_{2}+2{\mathrm{OH}}^{-}$$

The production of OH^−^ ions by the cathodic reactions (1) and/or (2) induces an increase of the interfacial pH that may lead to the formation of a calcareous deposit [16,17,18] on the steel surface or even promote the passivation of the metal surface [19] or the formation of a protective oxide layer [20]. In the field, the aimed applied potential is lower than the threshold of −0.85 V vs. Cu/CuSO_4_, typically about −0.95 to −1.0 V vs. Cu/CuSO_4_. Significantly lower potentials, e.g., as −1.2 V vs. Cu/CuSO_4_, are avoided because a high cathodic rate associated with intense water reduction leads to a very high interfacial pH. This can be detrimental for the coating associated with CP to protect the metal from corrosion (as recalled in the corresponding standard [14]) because it can generate a delamination process.

Previous studies were devoted to the effectiveness of cathodic protection (CP) of buried steel structures and/or to its failure mechanisms under different soil conditions [21,22]. Various factors were reported to have a significant influence towards CP effectiveness, in particular those affecting the transport of crucial components (O_2_, OH^−^, etc.). Such factors are, for instance, the coating defect size and the properties of the environment surrounding the protected object [21,22]. CP modifies the chemical environment of the metal, mostly because of the accelerated cathodic processes [21,22]. Consequently, the kinetics and mechanism of the metal surface corrosion change, and all these changes depend on the applied cathodic potential. It proved necessary to determine the residual corrosion rate of the buried carbon steel in different kinds of soil that would, in turn, quantify the CP effectiveness and its link with the applied potential. Barbalat et al. [23,24] developed a method based on voltammetry to estimate the instantaneous residual corrosion rate of carbon steel in soil in the presence of CP. The methodology was optimized afterwards using a computer-fitting procedure of the polarization curves achieved using electrochemical laws [25,26]. 

Besides, results obtained with electrochemical impedance spectroscopy (EIS) demonstrated that in unsaturated soils (about 35–65% saturation) CP could lead to an increase of the active (or “wet”) area [25,26], a phenomenon more likely due to electrocapillary effects. In an unsaturated soil, the “wet” area is the part of the steel surface in contact with the soil electrolyte. The rest of the surface, which is in contact with air, does not participate in the electrochemical processes. More recently, the combined effects of cathodic polarization and variations of soil humidity were studied and it was shown that these effects depended on the applied potential [27]. It was also observed that the residual corrosion process led to a porous magnetite layer that influenced the cathodic reaction [27]. 

In some cases, the decrease of the potential indeed led to a decrease of the residual corrosion rate [20,27]. In other cases, an opposite effect was observed, suggesting that an excessively cathodic potential could be detrimental, more likely by hindering the formation of a protective layer of (residual) corrosion products [24,25]. 

The present study was then designed to monitor, using voltammetry and EIS, the evolution of the steel/electrolyte interface during “excessive” and “normal” CP in an unsaturated soil. More precisely, the main aim was to compare the protective ability of the mineral layer forming on the steel surface in both situations. For that purpose, steel coupons were buried in an artificial unsaturated soil for 90 days. They were left at open circuit potential (OCP) for the first seven days before CP was applied for the remaining 83 days of the experiment. EIS measurements were performed to study the evolution of the steel/electrolyte interface, while voltammetry was used to determine the residual corrosion rate using the methodology developed in previous works [25,26,27]. The characterization of the mineral layer covering the coupons at the end of the 90-day experiment was achieved by µ-Raman spectroscopy.

## 2. Materials and Methods

### 2.1. Preparation of Steel Coupons

This study made use of carbon steel S235JR as the testing material because it is commonly used for gas pipeline transportation system [28]. The nominal chemical composition of this material is (in wt.%): C: 0.17% max., Mn: 1.40% max., P: 0.035% max., Cu: 0.55% max, N: 0.012% max., and Fe for the rest. Cylindrical coupons of diameter 30 mm and 2 mm thickness were cut from the same carbon steel rod. The cut carbon steel coupons were connected to a copper wire and embedded in resin. The carbon steel coupons were mechanically prepared through grinding at 80 to 600 grits (grain size 26 µm). After grinding, the coupons were rinsed thoroughly with Milli-Q water and dried rapidly with a hairdryer. Only one circular side of the coupon (corresponding to the surface area of 7.07 cm^2^) was exposed to the soil environment.

### 2.2. Soil Preparation and Electrochemical Cell Set Up

This study made use of an artificial soil composed of 83 wt.% fine sand (SiO_2_) particles (average particle size 22 µm), 14.5 wt.% clay (kaolinite) and 2.5 wt.% peat. The soil environment used for all the experiments was prepared by incorporating and mixing the electrolyte solution and the artificial soil to achieve ~65% soil saturation level (65% sat.). The selection of the electrolyte shown in Table 1 was mainly based on trying to achieve the solution with species commonly present in soil, particularly Ca^2+^ and Mg^2+,^ which may play a role during cathodic protection, e.g., by inducing the formation of a calcareous deposit. The resulting pH, once soil and electrolyte were mixed, was measured at 7.1 ± 0.1. All experiments were performed at room temperature (21 ± 2 °C). 

**Figure 1:**
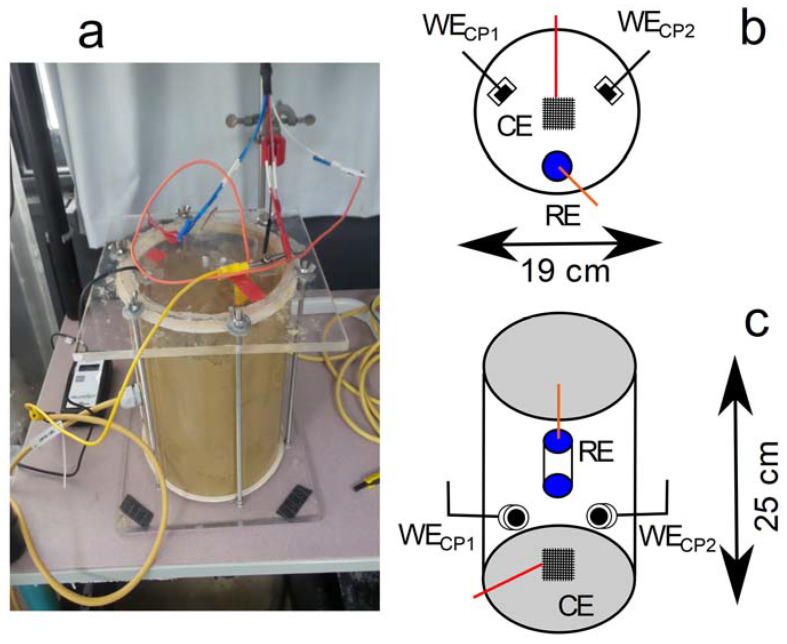
Representation of electrochemical cell for corrosion studies in soil: (**a**) picture of the cell, (**b**) schematic top view and (**c**) schematic side view. CE = counter electrode, RE = reference electrode, WE_CP1_ = working electrode cathodic protection 1 (steel coupon 1) and WE_CP2_ = working electrode cathodic protection 2 (steel coupon 2). The moisture sensor was omitted for clarity; the sensor reader is visible on the left of the picture.

A 19 cm × 25 cm Plexiglas cell (Figure 1) was used to bury two coupons inside the prepared soil, ensuring that they were as far apart from each other as possible. The soil was filled up to about 5–6 cm below the top of the cell to allow the remaining space for air (i.e., O_2_) into the system. The cell was closed with a lid to maintain constant soil moisture. The prepared steel coupons were used as working electrodes. The reference electrode was the Celco 5 (COREXCO, Décines Charpieu, France) copper-copper sulfate (Cu/CuSO_4_) electrode (+0.316 V/SHE at 25 °C) specifically designed for soil experiments and commonly used in the field. It was also used in previous studies [10,11,23,24,25,26,27]. All electrochemical potentials were expressed with reference to this electrode, i.e., V vs. Cu/CuSO_4_. A titanium grid, placed approximately at the center of the cell, was used as a counter electrode. Soil moisture was controlled during the experiment with a Waterscout SM100 sensor (Spectrum Technologies Inc., Aurora, IL, USA). It proved constant at 63 ± 3% sat. 

The two carbon steel coupons, denoted as WE_CP1_ and WE_CP2_, were buried in soil for 90 days. They were left at OCP for the first 7 days of the experiment. This duration was assumed sufficient for the system to reach a steady state, and the corrosion rate was then estimated by electrochemical measurements at day 7. CP was subsequently applied on the coupons for the remaining 83 days of the experiment. 

### 2.3. Electrochemistry

#### 2.3.1. Cathodic Protection

After the first 7 days at OCP, CP was applied on coupons WE_CP1_ and WE_CP2_ at potential values, corrected from ohmic drop, *E*_CP_ = −1.0 and −1.2 V vs. Cu/CuSO_4,_ respectively. EIS measurements (see next section) were performed at 24-h time intervals to determine the soil electrolyte resistance *R*_s_. The applied potential was then corrected from ohmic drop according to the expression:

(3) $$E_{\mathrm{CP}}=E_{IRfree}=E_{on}-R_{s}\cdot I$$

where *E*_IRfree_ is the corrected potential, *E*_on_ is the applied potential, *R*_s_ is the soil electrolyte resistance and *I* is the current flowing through the electrode. Because *I* may vary with time, *E*_CP_ may also vary until another correction is made (next day).

#### 2.3.2. Electrochemical Impedance Spectroscopy (EIS) Experiments 

All electrochemical measurements were performed in the cell displayed in Figure 1 using Gamry Interface 1000 potentiostats (Gamry Instruments, Warminster, PA, USA) monitored by the Framework software (V4.35, Gamry Instruments, Warminster, PA, USA). The results obtained were analyzed with the Gamry Echem Analyst software (V6.31, Gamry Instruments, Warminster, PA, USA). Electrochemical impedance spectroscopy (EIS) measurements were performed at a frequency between 100 kHz and 200 mHz, and the AC voltage perturbation amplitude (peak to peak) was 0.03 V around the applied protection potential of the sample (for coupons under CP) or around OCP (before the CP was applied). This high amplitude was required because of the important ohmic drop due to soil resistivity. The linearity of the system was checked in varying the amplitude of the AC signal applied to the sample. 

The soil electrolyte resistance *R*_s_ can be determined by EIS. It corresponds to the real part of the impedance when the frequency tends towards infinite. It must be noted that *R*_s_ is mainly linked, in unsaturated soils, to the “wet” area of the electrode [10,11,25,26], i.e., the area really in contact with the electrolyte present in the pores of the soil. More precisely, *R*_s_ varies inversely to the wet area and, for instance, if the soil dries, i.e., the amount of electrolyte present in the pores decreases, then the wet area decreases and *R*_s_ increases. The value of *R*_s_ can then indicate if soil moisture varies at the vicinity of the coupon [11].

Actually, important variations of *R*_s_ were observed between day 7 (beginning of CP) and day 42. This rather corresponds to a transition period between steady state OCP conditions and steady state CP conditions (this is discussed in Section 3.1). For this reason, it was decided to focus the present work on the characterization of the system after day 42. 

#### 2.3.3. Voltammetry Experiments

For the coupons at OCP during the 7 first days, the polarization curves were recorded at a scan rate (d*E*/d*t*) of 0.2 mV/s from OCP up to +0.08 V and down to −0.08 V, i.e., on a limited range of potential so that the steel surface was only moderately affected. This method was used previously and referred to as “VAOCP” for “voltammetry around OCP” [10,29]. It implies that the voltammograms obtained are mathematically modeled using electrochemical kinetic laws, which was achieved using the OriginPro 2016 Software (SR0 b9.3.226, OriginLab Corporation, Northampton, MA, USA). Electrochemical parameters such as anodic and cathodic Tafel coefficients are then obtained together with the corrosion current density *j*_corr_. The corrosion rate *τ*_corr_ is then computed from *j*_corr_ using Faraday’s law. 

For coupons under CP, the polarization curves were recorded from the applied potential *E*_CP_ up to (approximately) OCP + 0.08 V, with d*E*/d*t* = 0.2 mV/s. They were modelled as described elsewhere [25,26,29], and as also detailed further in the text (Section 3.2), to estimate *τ*_rc_, the “residual” corrosion rate. *τ*_rc_ is actually the corrosion rate associated with the anodic current density *j*_A_ at the applied potential *E*_CP_ [23,24,25,26], i.e., *j*_A_(*E*_CP_). Because voltammetry may affect significantly the behavior of the system, the first polarization curves were acquired at day 42, and only once a week afterwards, except at day 56, i.e., at days 49, 63, 70, 77, 84 and 90. During the first 35 days of CP (from day 7 to day 42), i.e., during the transition period, the system was thus only subjected to minor perturbations, corresponding to EIS measurements. 

### 2.4. µ-Raman Spectroscopy

At the end of the experiment, the coupons were removed from the electrochemical cell with a part of soil covering their surface and kept inside a freezer where they were maintained at −24 °C. This experimental procedure was used in previous works and proved adequate to preserve the system from further corrosion and evolution [30,31].

µ-Raman spectroscopy analysis was performed at room temperature with a Jobin Yvon High Resolution Raman Spectrometer (LabRAM HR, Horiba, Tokyo, Japan) equipped with a microscope (Olympus BX 41, Olympus, Tokyo, Japan), a Peltier-based cooled charge coupled detector (CCD) and a He-Ne laser (632.8 nm). The laser power was kept at 10% of the maximum (i.e., 0.9 mW) to prevent an excessive heating that can induce the transformation of the analyzed compounds into hematite (α-Fe_2_O_3_). The acquisition time was variable but equal to 60 s in most cases. It could be increased up to 5 min to optimize the signal to noise ratio. At least 20 zones (diameter of ~6 µm) of each sample were analyzed through a 50× objective, with a resolution of 0.1 cm^−1^.

## 3. Results

### 3.1. Electrochemical Impedance Spectroscopy

EIS measurements were performed on both coupons at various exposure times in soil at OCP or under CP. In the present case dealing with a resistive medium, a parasitic influence of the reference electrode at high frequency was observed [32], and *R*_s_ was then determined at 10 kHz, i.e., *R*_s_ = Re(*Z*) at 10 kHz. For the same reason the EIS data obtained at frequencies higher than 10 kHz were not considered and are not shown on the Nyquist plots except when noted. 

**Figure 2:**
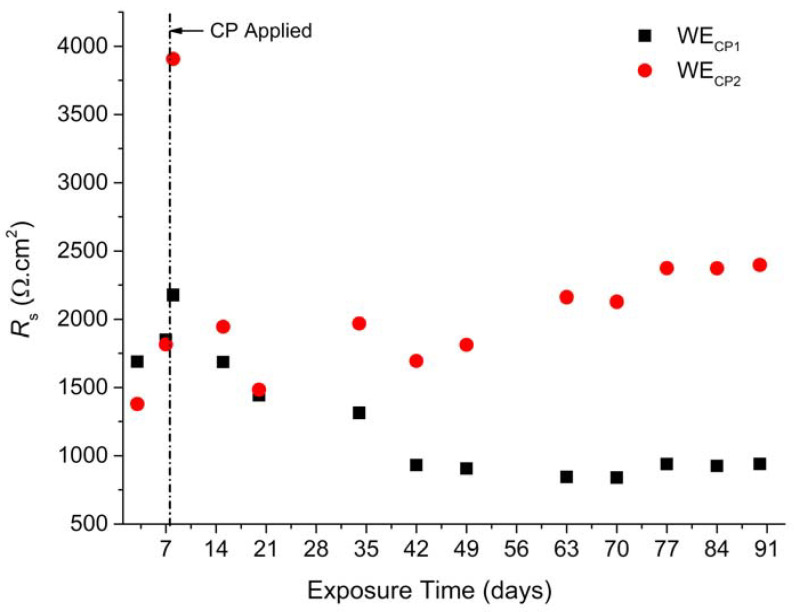
*R*_s_ evolution with exposure time for coupons WE_CP1_ and WE_CP2_.

First, Figure 2 displays the evolution of *R*_s_ with time for coupons WE_CP1_ and WE_CP2_ in soil during the whole experiment. During the OCP period (days 1–7), *R*_s_ increases from 1670 to 1865 Ω cm^2^ for coupon WE_CP1_ and from 1380 to 1815 Ω cm^2^ for coupon WE_CP2_. This increase in *R*_s_ can be attributed to the changes in soil physicochemical properties. Actually, water tends to flow vertically in the cell, because of gravity, so that a gradient of soil moisture appears with time. This movement of fluid necessarily modifies the soil at the vicinity of the coupons during the first days. Moreover, corrosion products are formed on the metal surface, which also modifies the steel/soil interface.

The application of CP leads to an immediate sharp increase in *R*_s_ to ~2250 Ω cm^2^ for coupon WE_CP1_ and ~3850 Ω cm^2^ for coupon WE_CP2_. Various changes occur at the steel/electrolyte interface when CP is applied: dissolved O_2_ is consumed and interfacial pH increases, the electric field induces migration of ions, electrocapillary effects modify the contact angle between liquid phase and metal, etc. It is worth noticing that there is a difference of 1600 Ω cm^2^ in *R*_s_ values obtained between coupons WE_CP1_ and WE_CP2_ after 8 days. This difference can then be attributed to the different applied *E*_CP_ values set for coupons WE_CP1_ and WE_CP2_, i.e., −1.0 and −1.2 V vs. Cu/CuSO_4,_ respectively. This indicates that lower *E*_CP_ leads to higher *R*_s_. This initial increase of *R*_s_ is, however, followed by a rapid decrease so that at day 14 both *R*_s_ values are similar to those measured at OCP. This initial increase of *R*_s_ is then a transient phenomenon that was not further studied and is yet to be explained.

However, clear trends are observed after day 21. For coupon WE_CP1_, *R*_s_ decreases further before reaching a constant value of 890 ± 50 Ω cm^2^ after day 42, indicating that a steady state has been reached. This value is actually ~800 Ω cm^2^ lower than the initial value of 1670 Ω cm^2^ (day 3). This result is consistent with previous study [25], where the decrease of *R*_s_ was attributed to an increase of the “wet area” due to electrocapillary effects. A negative shift of potential allows the liquid phase to spread over the steel surface because it decreases the solid–liquid contact angle *θ* [33,34,35,36].

In contrast, *R*_s_ values obtained for coupon WE_CP2_ after 21 days take an opposite trend in relation to that observed for coupon WE_CP1_, i.e., increase again with exposure time. This increase is not continuous, and some fluctuations are observed, with local minima at days 42 and 70. *R*_s_ reaches a final value of ~2400 Ω cm^2^ after 90 days, thus being ~1000 Ω cm^2^ higher than the initial value of 1380 Ω cm^2^ (day 3). At the CP potential of −1.2 V vs. Cu/CuSO_4_, the cathodic process is enhanced, leading to the acceleration of O_2_ consumption at the steel/electrolyte interface and more likely to the increase of water reduction rate. The increase of *R*_s_ with time may then be due to the accumulation of hydrogen (H_2_) bubbles that form on the steel surface according to Equation (2). This phenomenon would indeed lead to a decrease of the “wet” area (i.e., the area in contact with the liquid phase) and thus to an apparent increase of *R*_s_ [10,25]. The cathodic processes taking place at −1.2 V vs. Cu/CuSO_4_ are studied and discussed in Section 3.2.

**Figure 3:**
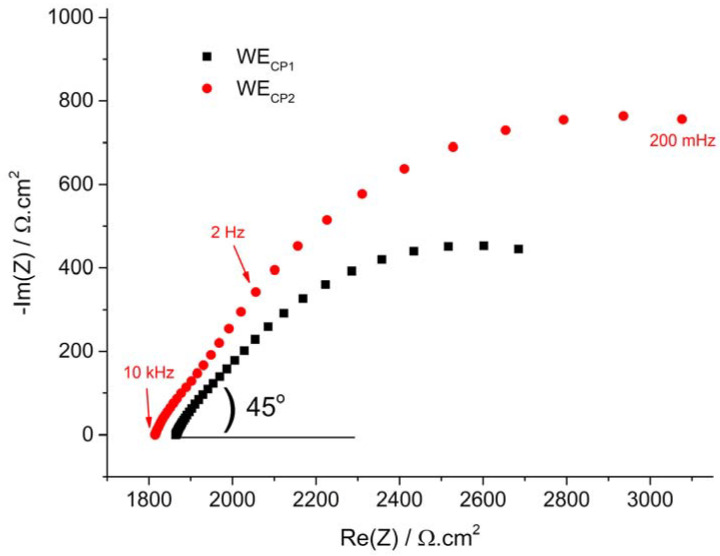
EIS Nyquist at OCP (day 7) for coupons WE_CP1_ and WE_CP2_.

The Nyquist EIS plots obtained for both coupons before the application of CP are compared in Figure 3. Both Nyquist diagrams display similar features, though they are more clearly seen for coupon WE_CP2_. A first capacitive loop is present at high frequency, followed by a linear part and a second capacitive loop at low frequency. The linear part makes angles of 45° and 50° with Re(*Z*) for coupons WE_CP1_ and WE_CP2,_ respectively. This shows the influence of a diffusional process. Previous studies reported that the cathodic reaction of the steel buried in unsaturated soil was indeed partially controlled by the diffusion of O_2_ [10,24]. Actually, the obtained Nyquist diagrams are characteristic of a bounded diffusion phenomenon, i.e., a finite-length diffusion for a planar electrode. At the beginning of the corrosion process, the corrosion product layer is very thin and should not hinder the diffusion of O_2_. The O_2_ concentration gradient is then located in the soil itself, as observed in previous work [37].

**Figure 4:**
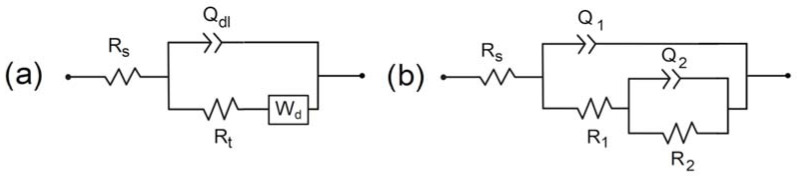
EEC used to fit the EIS data obtained at OCP (**a**) or under CP (**b**) for both coupons.

The EIS data at OCP were then modelled using the electrical equivalent circuit (EEC) displayed in Figure 4a. In this EEC, *R*_s_ is the soil electrolyte resistance, *R*_t_ is the charge transfer resistance, *Q*_dl_ a constant phase element (CPE) used to represent the double layer capacitance and *W*_d_ is a bounded diffusion impedance. The CPE was used instead of an ideal capacitance because the first attempts to model the experimental data, performed with a capacitance, did not lead to acceptable goodness-of-fit. Actually, CPE is commonly used because it takes into account various effects due to inhomogeneity, porosity, roughness and other non-ideal dielectric properties of the electrode [38]. The results obtained via this modelling are discussed together with those obtained for the coupons under CP at the end of this section.

**Figure 5:**
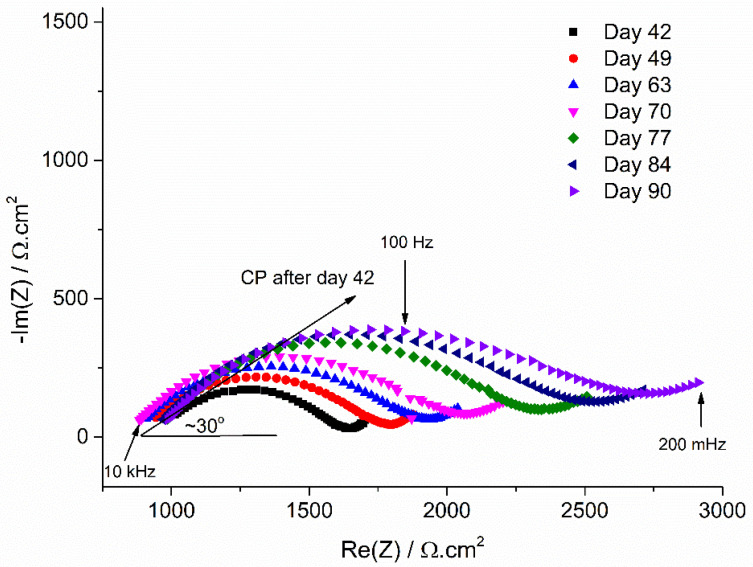
Evolution of the EIS Nyquist plots with exposure time for coupon WE_CP1_ (*E*_CP_ = −1.0 V vs. Cu/CuSO_4_) between days 42 and 90.

**Figure 6:**
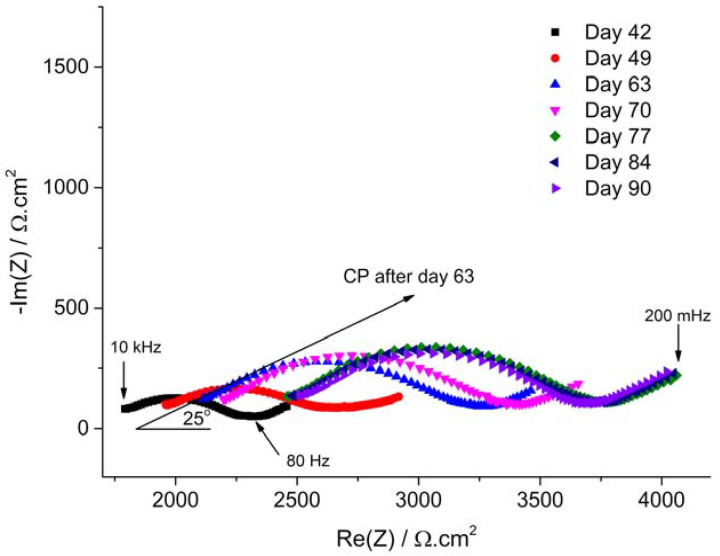
Evolution of the EIS Nyquist plots with exposure time for coupon WE_CP2_ (*E*_CP_ = −1.2 V vs. Cu/CuSO_4_) between days 42 and 90.

The influence of CP can be observed on the Nyquist plots of Figure 5 and Figure 6. As explained in Section 2.3.2, only the data obtained after day 42 are discussed. These Nyquist diagrams consistently exhibit a flattened semi-circle loop, followed by what may be an incomplete second loop at lower frequency. The main flattened semi-circle loop is characterized, like that observed at OCP, by a linear behavior at high frequency. However, the initial linear part of the Nyquist diagrams obtained under CP after day 42 makes an angle of 30° and 25° with Re(*Z*) for coupons WE_CP1_ and WE_CP2,_ respectively, as illustrated on day 42 Nyquist plot in Figure 5 and day 63 Nyquist plot in Figure 6. This suggests that the steel coupons behave as semi-infinite porous conductive electrodes [37,39,40]. The cathodic process, predominant under CP, would then involve the diffusion of O_2_ inside the pores of a conductive mineral film covering the steel surface and its reduction all along the conductive walls of the pores. At OCP, the initial linear part of the Nyquist diagram made an angle of about 45° with the Re(Z) axis (Figure 3). The impedance, mainly corresponding to a bounded diffusion impedance, then described the diffusion rate of oxygen through a non-conducting porous layer, which in the present case was more likely the soil itself. At day 42, i.e., after 35 days under CP, the impedance describes the diffusion (and reduction) of O_2_ in the pores of a conductive layer that formed later under CP.

Furthermore, the influence of the applied potential is also clearly revealed. In the case of the CP potential of −1.0 V vs. Cu/CuSO_4_, i.e., coupon WE_CP1_ as shown in Figure 5, the flattened semi-capacitive loop at high frequency of the Nyquist plots is observed to increase in magnitude with exposure time. In the present case, the electrochemical process is mainly controlled by the diffusion of O_2_ inside the pores of the mineral film covering the steel surface. Consequently, the gradual increase of the resistance associated with this electrochemical process indicates that the mineral film hinders more and more efficiently the diffusion of O_2_.

In the case of excessive CP, i.e., coupon WE_CP2_ as shown in Figure 6, the evolution of the Nyquist plots with exposure time is significantly different. The main difference relates to the evolution of *R*_s_, whose value increases with time in this case (see also Figure 2). The diameter of the main flattened loop increases with time between day 42 and day 63, as observed for coupon WE_CP1_, but does not seem to increase afterwards.

**Figure 7:**
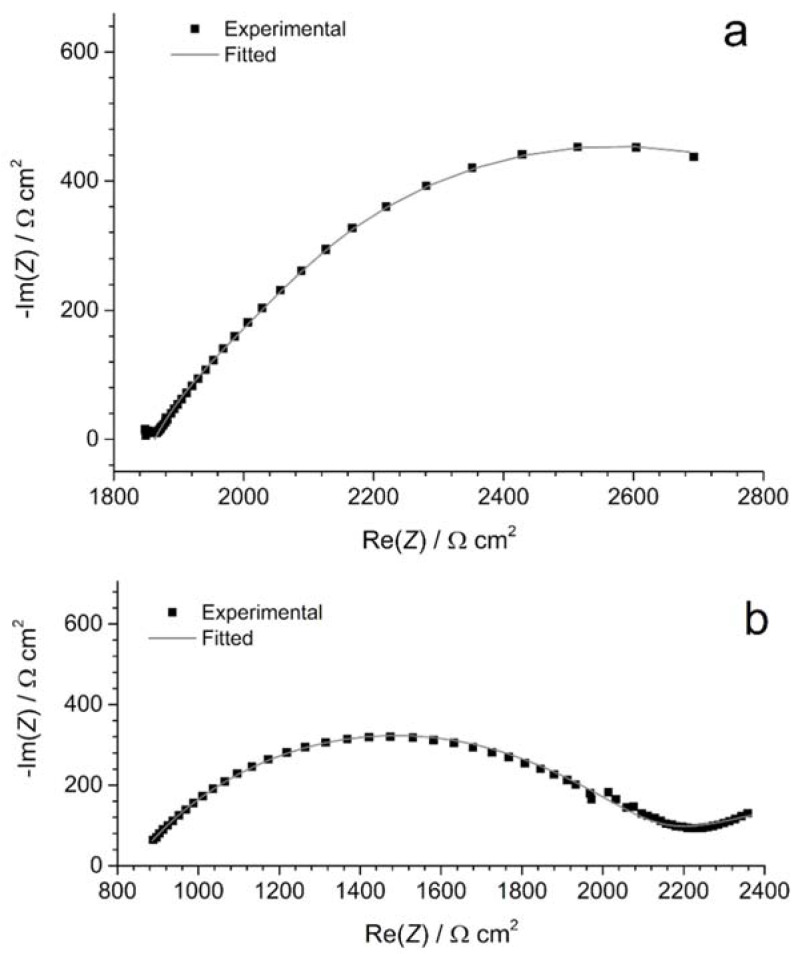
EIS modelling: Nyquist plots for coupon WE_CP1_ (**a**) at OCP at day 7 and (**b**) under CP at day 70. Owing to a lower *R*_s_ value, the data obtained at day 70 were usable from 100 kHz to 100 mHz.

To obtain additional information and quantify the evolution of the steel/soil interface with time, a modelling of the EIS data obtained for the coupons under CP was achieved. The EEC used for both coupons is displayed in Figure 4b. In this EEC, *Q*_1_ and *R*_1_ are a CPE and a resistance, respectively, used to model the phenomenon associated with the main flattened capacitive loop present in all cases (Figure 5 and Figure 6). *Q*_2_ and *R*_2_ are the elements used to fit the low frequency part of the impedance diagram. Because only a very small part of the corresponding capacitive loop (if one assumes this is really a capacitive loop) is seen, various values could be obtained for *Q*_2_ and *R*_2_ parameters and consequently these parameters are not presented nor discussed. Various fits were performed, with different values for *R*_2_ and *Q*_2_, to study the influence of these parameters on the values obtained for *R*_1_ and *Q*_1_. It was noted that *R*_1_ and *Q*_1_ were not significantly influenced by *R*_2_ and *Q*_2_. The considered EEC led to satisfactory fittings of the experimental data, as illustrated by Figure 7.

Figure 7a shows as an example the Nyquist plot for coupon WE_CP1_ at OCP (day 7) and Table 2 gathers the results obtained for both coupons at OCP. It can be seen that the numerical values are similar for both coupons, except for resistance *R*_d_. In the expression of the bounded diffusion impedance *W*_d_, *R*_d_ is a scaling factor that depends on the kinetics of the interfacial reaction and the bulk concentration of the electroactive species. The larger value of *R*_d_ for coupon WE_CP2_ suggests a lower O_2_ concentration. This would indicate a faster O_2_ consumption at the vicinity of coupon WE_CP2_. This faster evolution may also correlate with the faster evolution of *R*_s_ observed for this coupon between day 1 and day 7 (Figure 2). In conclusion, both coupons behave quite similarly at OCP, as confirmed by voltammetry measurements (Section 3.2).

Figure 7b shows as an example the Nyquist plot for coupon WE_CP1_ under CP at day 70, and Table 3 gathers the results obtained between days 42 and 90 for both coupons. First, as observed qualitatively using the Nyquist plots of Figure 5 and Figure 6, the resistance *R*_1_ associated with the main capacitive loop increases continuously with time for coupon WE_CP1_, to reach a maximum of 1661 Ω cm^2^ at day 90. For coupon WE_CP2_, after an initial increase between days 42 and 63, where a maximum of 1334 Ω cm^2^ is reached, *R*_1_ decreases slightly to end at 1230 Ω cm^2^.

It is interesting to note that the *n* coefficient of the CPE is low, between 0.52 and 0.60 considering both coupons. This coefficient measures the deviation from an ideal behavior, i.e., *n* = 1 for a true capacitor and *n* < 1 for a CPE. However, a “real” CPE is characterized by *n* values higher than 0.7, and *n* = 0.5 corresponds to a diffusion element. The values of *n* obtained here in each case confirm that the cathodic process, i.e., mainly oxygen reduction, is strongly influenced by diffusion. Parameter *R*_1_ can then be considered as a “resistance to diffusion” induced by the mineral layer growing on the steel surface and inside the pores of the soil. For coupon WE_CP1_, the continuous increase of this resistance shows that this layer is more and more protective as it grows with time. Simultaneously, the CPE element *Q*_1_ also increases continuously with time. It was observed that the Nyquist plots corresponded to a porous conductive electrode behavior, more likely due to the formation of a porous magnetite layer [20,27]. The growth of this conductive layer would increase the overall cathodic surface, thus leading to an increase of the associated capacitance.

Moreover, the increase of *R*_1_ for coupon WE_CP1_ is not associated with an increase of *R*_s_, which indicates that this increased “resistance to diffusion” is not in any case associated with a decrease in the active area of the electrode. It can really be attributed to the growth of the porous conductive layer that covers the steel surface and progressively hinders more and more the transport of O_2_.

In the case of coupon WE_CP2_, the initial increase of *R*_1_ is associated with an increase of *R*_s_ that may be due to a decrease of the active area [25]. However, *R*_s_ increases from 1711 Ω cm^2^ to 2142 Ω cm^2^ between day 42 and day 63, i.e., an increase of +25%, while *R*_1_ increases from 595 Ω cm^2^ to 1334 Ω cm^2^, i.e., an increase of +124%. Consequently, the main reason for this initial increase of *R*_1_ is not the possible decrease of the active area but, as for coupon WE_CP1_, a real increase of the “resistance to diffusion” associated with the growth of the porous conductive layer. However, *R*_1_ decreases slightly after day 63, while *Q*_1_ decreases as soon as day 42 and fluctuates after day 63. This shows that the protective ability of the porous conductive layer is influenced by a phenomenon that does not take place for coupon WE_CP1_. This could be due to the hydrogen evolution reaction that should be more important at the lower potential of −1.2 V vs. Cu/CuSO_4_. Hydrogen evolution could damage, through the release of H_2_ bubbles, the porous conductive layer. This point is discussed further at the end of Section 3.3.

### 3.2. Voltammetry and Estimation of Residual Corrosion Rate

The voltammograms obtained in this study were computer fitted using theoretical kinetic laws. From the EIS experiments, discussed in the previous section, it was observed that the cathodic process, mainly linked to O_2_ reduction, was at least partially controlled by diffusion. Therefore, the mathematical modelling of the voltammograms *j*(*E*) used in the present study, adapted from that reported previously [10,11,25,27], was described by the following expression:

(4) $$j=j_{A}+j_{C}=j_{corr}\cdot e^{\beta_{A}\left(E-E_{corr}\right)}+\frac{1}{\frac{1}{j_{lim}}-e^{-\beta_{C}\left(E-E_{corr}\right)}\cdot \left(\frac{1}{j_{corr}}+\frac{1}{j_{lim}}\right)}$$

where *j* = overall current density, *j*_A_ = anodic current density, *j*_C_ = cathodic current density, *j_c_*_orr_ = corrosion current density, *j*_lim_ = limiting current density (O_2_ diffusion) in A/cm^2^, *E*_corr_ = corrosion potential in V vs. Cu/CuSO_4_, *β*_A_ = anodic Tafel coefficient and *β*_C_ = cathodic Tafel coefficient for O_2_ reduction in V^−1^. 

This model corresponds to an anodic process controlled by charge transfer and a cathodic process partially controlled by diffusion (mixed control) [10,24,25,29]. The cathodic process is then in this case restricted to O_2_ reduction. An additional contribution due to water reduction was envisioned. However, this model implied a very large number of adjustable parameters and the procedure proved unreliable as various solutions could be obtained for the fitting of the experimental curves. It was consequently discarded.

**Figure 8:**
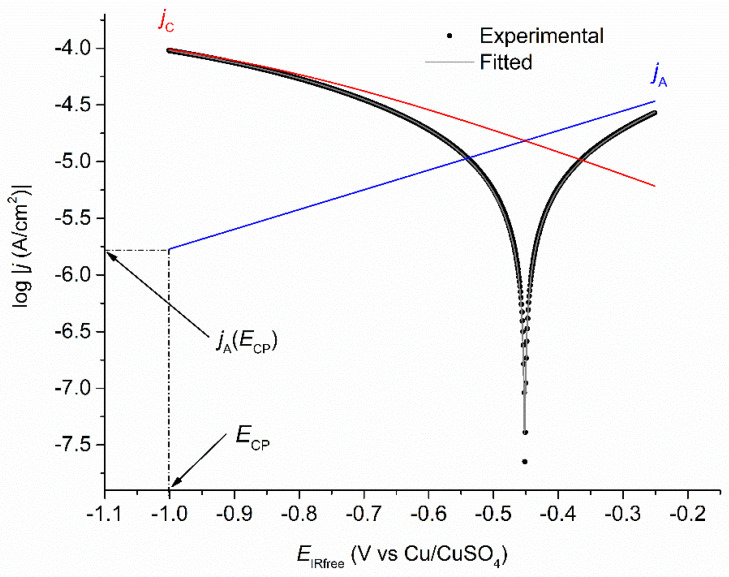
Polarization curves for coupon WE_CP1_ at day 49: experimental-curve (black dots), fitted-curve (grey full line), anodic (blue line) and cathodic (red line) components drawn using the obtained electrochemical parameters.

Equation (4) was then used in any case to perform the mathematical modelling, and Figure 8 shows as an example the computer fitted log|*j*| vs. *E* voltammogram obtained after 49 days for coupon WE_CP1_. In any case, the quality of the fitting proved excellent, which demonstrates that the electrochemical model was adequate. Actually, at *E*_CP_ = −1.0 V vs. Cu/CuSO_4_, it was indeed expected that the contribution of water reduction was negligible. The current density *j*_A_(*E*_CP_) was extrapolated from the anodic Tafel line log|*j*_A_| vs. *E*, as illustrated in Figure 8. From the extrapolated *j*_A_(*E*_CP_) value, the residual corrosion rate *τ*_rc_ was estimated via Faraday’s law.

All the fitted parameters for coupon WE_CP1_ are listed in Table 4. To facilitate comparison with published data, the Tafel coefficients *β_A,C_* were converted to Tafel slopes *b_A,_*_C_ using the expression:

(5) $$b_{A,C}=\frac{\ln\left(10\right)}{\left|\beta_{A,C}\right|}$$

The accuracy of the obtained values is about ±10% in any case. For *E*_corr_, determined for coupon WE_CP1_ directly on the graph and not through the fitting procedure, the accuracy is ±1 mV. 

Firstly, the voltammetry measurements performed on day 7 just before the application of CP revealed that the corrosion rate *τ*_corr_ was 330 µm yr^−1^. This value is consistent with that obtained for a similar saturation level (~70–60% sat.) in a previous work performed in the same artificial soil [10]. After 42 days of experiment (35 days of CP), the residual corrosion rate *τ*_rc_ of coupon WE_CP1_ was estimated at 19 µm yr^−1^. It decreased to 7 µm yr^−1^ after 77 days in soil (70 days of CP) and remained constant until the end of the experiment.

Table 4 also shows that under CP, *E*_corr_ increased from −0.54 vs. Cu/CuSO_4_ after 42 days in soil (35 days of CP) to a final value of −0.36 V vs. Cu/CuSO_4_ after 90 days in soil (83 days of CP). These values are much higher than the initial *E*_corr_ value measured at OCP. It illustrates the major changes induced by CP at the steel/electrolyte interface. The main change is the increase in interfacial pH, which itself can induce other effects, e.g., calcareous deposition or passivation of the steel surface.

The differences observed between the Tafel coefficients measured at OCP and those obtained when the coupon is under CP also reveal the strong influence of CP on the steel/soil interface. The application of CP actually led to a significant increase of *b*_A_ from 52 mV/decade at OCP to an average value of 440 mV/decade under CP. Similarly, *b*_C_ increased from 62 mV/decade to the same average of 440 mV/decade. This phenomenon was already observed in a previous study [27] conducted in similar soil environment and was attributed to the presence of a magnetite layer. The anodic reaction could involve the Fe(II,III) cations inside the magnetite layer and the dissolution of the oxide, thus varying *b*_A_. Similarly, *b*_C_ could vary because O_2_ reduction can take place on the magnetite layer and not necessarily on the steel surface. Finally, the limiting current density |*j*_lim_| seems to have decreased slightly with time as the larger (absolute) values are observed at days 42–63 and the lower (absolute) values at days 70–90.

**Figure 9:**
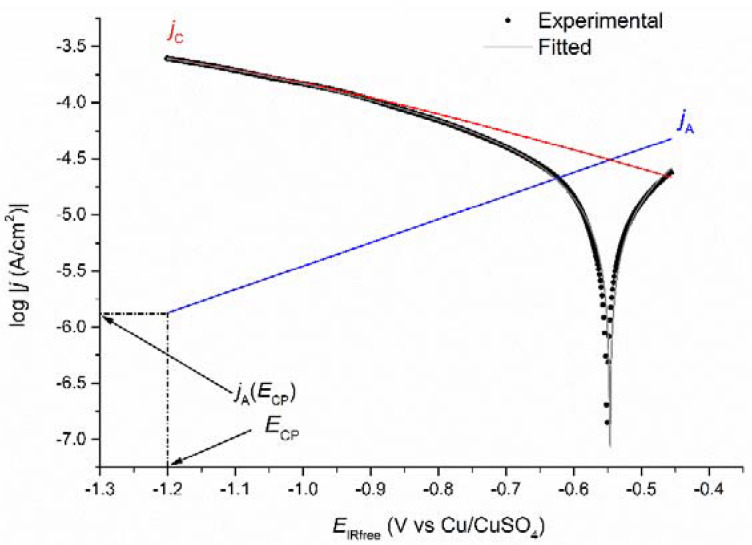
Polarization curves for coupon WE_CP2_ at day 49: experimental-curve (black dots), fitted-curve (grey full line), anodic (blue line) and cathodic (red line) components drawn using the obtained electrochemical parameters.

For coupon WE_CP2_ polarized at a lower potential of −1.2 V vs. Cu/CuSO_4_, the influence of water reduction could be significant. However, O_2_ reduction seems to be predominant because the EIS data, similar to those obtained for coupon WE_CP1_, revealed the importance of a diffusional process. As explained above, due to an excessive number of adjustable parameters, it proved unreliable to add another cathodic process to the modelling. Therefore, the voltammograms obtained for coupon WE_CP2_ were also computer-fitted using Equation (4). Acceptable fittings were achieved, as illustrated in Figure 9 with the voltammogram obtained after 49 days in soil.

**Figure 10:**
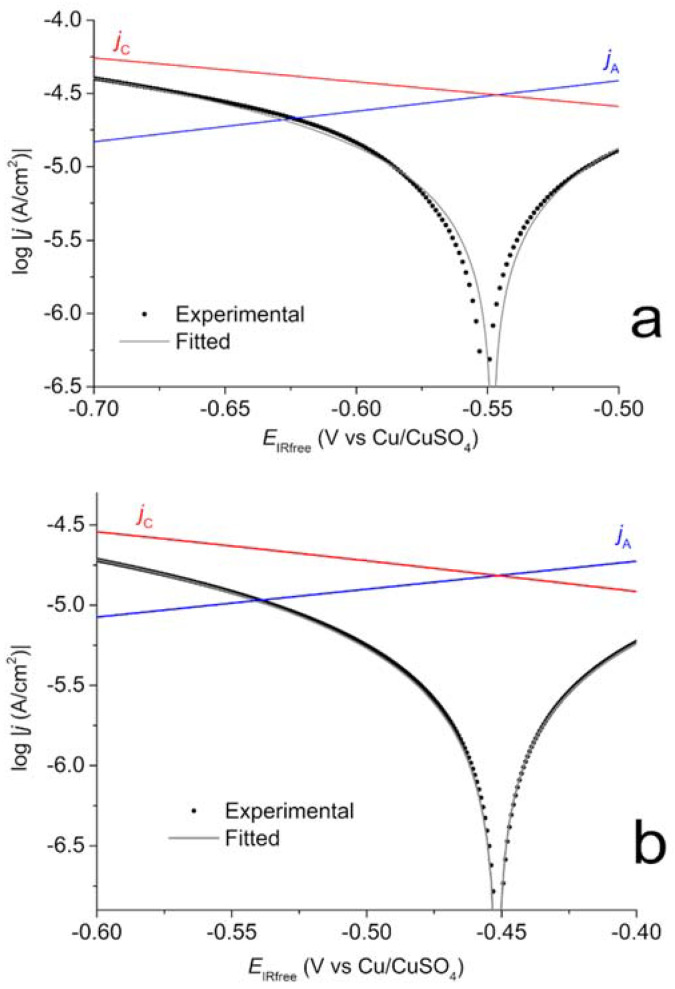
Polarization curves for coupons WE_CP2_ (**a**) and WE_CP1_ (**b**) at day 49: detailed view. Experimental-curve (back dots), fitted-curve (grey full line), anodic (blue line) and cathodic (red line) components drawn using the obtained electrochemical parameters.

The quality of the fittings was, however, not as good as that achieved for coupon WE_CP1_. In Figure 9, it can be seen that a slight shift of *E*_corr_ proved necessary to obtain this fitting. Figure 10 shows a focus on the zone where the discrepancy between experimental and fitted curves is the highest. Figure 10a relates to coupon WE_CP2_ and shows some important discrepancies around *E*_corr_ and in the *E*_IRfree_ region extending from −0.60 to −0.65 V vs. Cu/CuSO_4_. Figure 10b relates to coupon WE_CP1_ and shows that the modelling is in this case excellent. This shows that the electrochemical model considered in both cases is completely valid for coupon WE_CP1_ but is only an approximation for coupon WE_CP2_. This can be attributed to a significant contribution of water reduction for WE_CP2_ polarized at −1.2 V vs. Cu/CuSO_4_.

The fitted parameters obtained for coupon WE_CP2_ are listed in Table 5. Due to the lower goodness-of-fit, the accuracy is in this case about ±20%, as high as ±50% for the parameters linked to the cathodic reaction, i.e., *b*_C_ and *j*_lim_. As already demonstrated in other sections of the study, the application of CP after the first 7 days of the experiment induced significant changes on the steel/electrolyte interface. The evolution of *E*_corr_, *b*_A_ and *b*_C_ observed for coupon WE_CP1_ are also observed for coupon WE_CP2_. *E*_corr_ is increased by the application of CP and reaches −0.369 V vs. Cu/CuSO_4_ at day 90. Tafel slopes *b*_A_ and *b*_C_ are also increased by CP, to average values of 510 mV/decade and 660 mV/decade, respectively. The standard deviation on the *b*_A_ values is 60 mV/decade (±12%), which shows that the accuracy of the anodic Tafel slope is correct, i.e., not too strongly influenced by the imperfect modelling of the cathodic reaction. This is a crucial point because the estimation of the residual corrosion rate is primary linked to the anodic Tafel slope *b*_A_. By comparison, the standard deviation for the *b*_C_ values is 285 mV/decade (±43%). The error on *j*_lim_ is also important, and the values vary around an average of −5 × 10^−4^, with no apparent link with the polarization time. This can also be attributed to the influence of water reduction.

**Figure 11:**
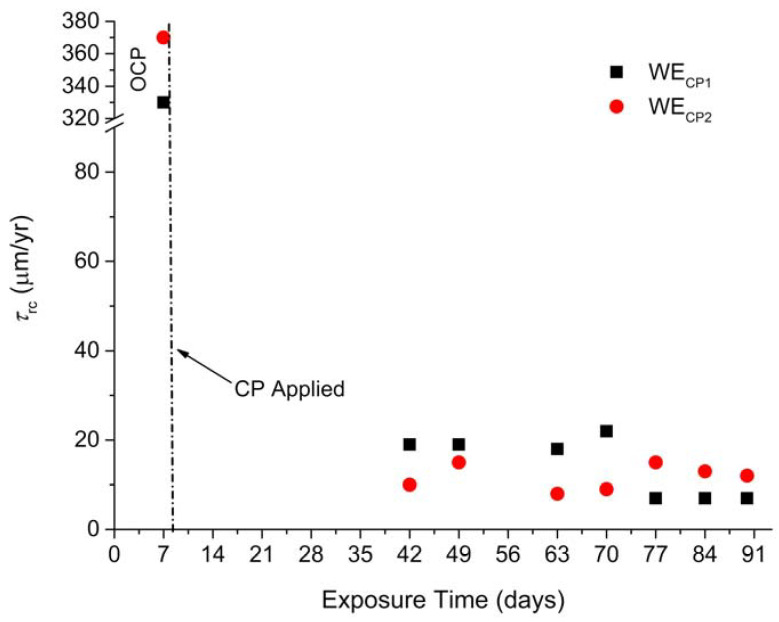
Evolution of the residual corrosion rate over time for coupons WE_CP1_ and WE_CP2_.

Similarly, the variations of the residual corrosion rate over time are different for WE_CP1_ and WE_CP2_. Figure 11 thus presents the evolution of *τ*_rc_ with exposure time in soil for both coupons. Firstly, it can be clearly observed that the application of CP led to a significant decrease in steel corrosion rate. Between days 42 and 70, the residual corrosion rate reaches values between 18 and 22 µm yr^−1^ for coupon WE_CP1_ and between 8 and 15 µm yr^−1^ for coupon WE_CP2,_ while the corrosion rates were estimated at 330 µm yr^−1^ and 370 µm yr^−1^, respectively, at OCP on day 7. This result is consistent with what could be expected: the lower the potential, the lower the anodic component of the overall current density (as illustrated by the anodic Tafel lines of Figure 8 and Figure 9).

After day 70, two opposite trends are observed. For coupon WE_CP1_, polarized at a correct protection potential, *τ*_rc_ decreases to reach a value below 10 µm yr^−1^, i.e., 7 µm yr^−1^ at days 77, 84 and 90. This decrease of the residual corrosion rate to such low values was already observed and was attributed to the progressive growth of a protective mineral layer on the steel substrate [23,24,25,26]. In contrast, for coupon WE_CP2_ polarized at a lower potential, *τ*_rc_ slightly increases after day 63, to reach 15 µm yr^−1^ at day 77 before to decrease slightly to 12 µm yr^−1^ at day 90. In conclusion, the average final (days 77–90) value is 7 µm yr^−1^ for coupon WE_CP1_ and 13 µm yr^−1^ for coupon WE_CP2_. The overprotection did not lead to an increased effectiveness of CP and seems to have even decreased this effectiveness. It can be assumed that the overprotection, which increases significantly water reduction and then hydrogen evolution, can damage the protecting mineral layer that covers the steel surface. This assumption also explains why, after an initial decrease to 8 µm yr^−1^, *τ*_rc_ increased later on.

Two different behaviors were also observed via EIS, in particular for the evolution of *R*_s_ over time (Figure 2). For coupon WE_CP1_, the *R*_s_ value remained constant after day 42, which indicates that the steel/soil interface has reached a steady state. Conversely, for coupon WE_CP2_, *R*_s_, after an initial decrease until day 21, tended to increase regularly with time. This increase of *R*_s_ could be associated with a slight decrease of the “wet” or “active” area of the steel electrode [10,11,25,26]. This decrease of the active area could be due to the accumulation of H_2_ bubbles on the surface. The release of such bubbles from time to time would then damage the protective film and lead to the observed increase of *τ*_rc_. 

Similarly, the diameter of the main capacitive loop present on the Nyquist diagrams, associated with the “resistance to diffusion” *R*_1_, was observed to increase continuously with time for coupon WE_CP1_ (Figure 5 and Table 4). This also illustrates the increasing protective ability of the mineral layer forming on the steel surface. The increase of the “resistance to diffusion” was less important in the case of coupon WE_CP2_ (Figure 6 and Table 4) and stopped after day 63 (it even slightly decreased). This result confirms the lesser protectiveness of the layer and is consistent with the increase of *τ*_rc_ observed after day 63.

It must finally be recalled that the method used here, based on voltammetry, may significantly modify the electrode surface and thus may introduce some side effects and bias. For instance, the steel surface may be passive at *E*_CP_, due to the increase of the interfacial pH, and depassivate during the voltammetry experiments. The extrapolation of the anodic Tafel line, which is actually obtained mainly from the potential region around *E*_corr_, would then lead to an overestimation of *τ*_rc_. This depassivation phenomenon may, however, take a time longer than that required for the voltammetry experiment. The time required for that depassivation once CP is interrupted was reported to vary with the size of the electrode, from 1 h for a 5 × 5 mm electrode to 13 h for a 30 × 30 mm electrode [19]. It may, however, depend on the nature of the soil, the composition of the electrolyte present in the soil, the moisture content, the applied potential, etc.

Voltammetry also involves a charging current due to capacitive effects, and the measured current is then the sum of the faradaic current and the charging current [41,42]. When the applied potential is increased linearly with time, at is the case for the polarization curves modelled to estimate the residual corrosion rate, the positive charge density stored at the metal surface increases. In this case, the charging current flows in the direction of the anodic faradaic current [42]. Then, the measured current is necessarily higher than the anodic faradaic current. Because the charging current is proportional to the scan rate [42], the discrepancy between both currents increases with the scan rate, and for that reason a small scan rate of 0.2 mV/s was considered. In any case, as demonstrated previously [26], these capacitive effects also lead to an overestimation of the residual corrosion rate. 

Increasing the potential from *E*_CP_ to OCP decreases the cathodic reaction rate and the O_2_ consumption rate so that the steel/soil interface may progressively be enriched in dissolved O_2_. This modification also leads to an additional increase in the corrosion rate, i.e., the anodic component of the current density, which leads to an overestimation of the residual corrosion rate. 

In conclusion, it must be noted that the method based on voltammetry more likely overestimates the residual corrosion rate. It may, however, give fruitful information when it is used to compare between two situations, for instance, two different applied potentials as done here. Moreover, the results given by voltammetry can be confronted to those given by EIS, and in the present case both methods led to consistent conclusions. Finally, when residual corrosion rates lower than 10 µm yr^−1^ are obtained using voltammetry, it can be reasonably assumed that the actual residual corrosion rate is indeed lower than this threshold of 10 µm yr^−1^, which is considered to define an “efficient” CP.

### 3.3. Characterization of the Mineral Layer Formed on the Steel Coupons

**Figure 12:**
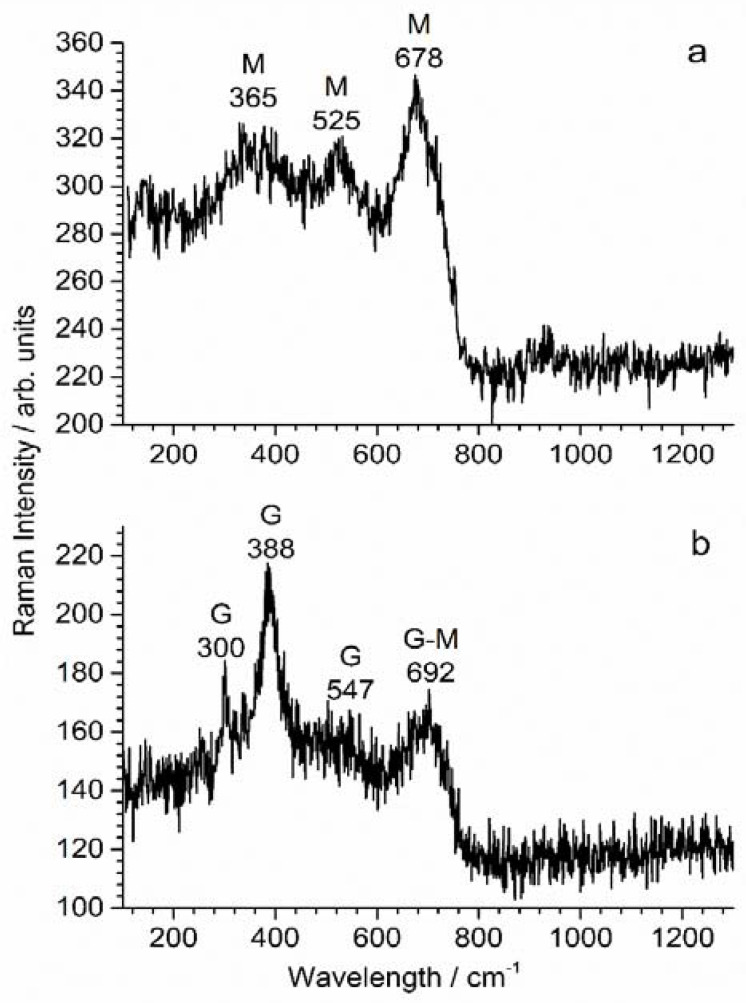
µ-Raman spectroscopy analysis of the surface of coupon WE_CP1_; G = goethite, M = magnetite/maghemite (see text). See text for the interpretation of spectra (**a**,**b**).

µ-Raman spectroscopy analysis was performed on the surface of the coupons and on the surface of the soil layer in contact with the steel surface. The same phases were identified in both cases, indicating that the corrosion products not only covered the steel surface but also tended to fill the pores of the soil. The analysis of coupon WE_CP1_ revealed various corrosion products, and two spectra are presented as examples in Figure 12. Spectrum (a) is very similar to that of magnetite (Fe_3_O_4_), which is characterized by an intense pic at 663–676 cm^−1^, and two smaller peaks at 300–320 cm^−1^ and 510–550 cm^−1^ [43,44,45]. However, in our case, the first peak is found at 365 cm^−1^ and its intensity, as well as that of the peak at 525 cm^−1^, is unusually strong. Actually, spectrum (a) also shares typical features with the spectrum of maghemite γ-Fe_2_O_3_. Magnetite and maghemite are structurally similar and may be both present. The formation of non-stoichiometric magnetite Fe_3-x_O_4_, an intermediate between Fe_3_O_4_ and γ-Fe_2_O_3_ [46], is also very likely. 

Raman spectrum (b) displays the main characteristic peaks of goethite (α-FeOOH) with an intense peak at 388 cm^−1^ and three smaller peaks at 300 cm^−1^, 547 cm^−1^ and 692 cm^−1^. The broad peak at 692 cm^−1^ is, however, unusually intense, which suggests that magnetite (main peak at 663–676 cm^−1^) is also present in this case.

**Figure 13:**
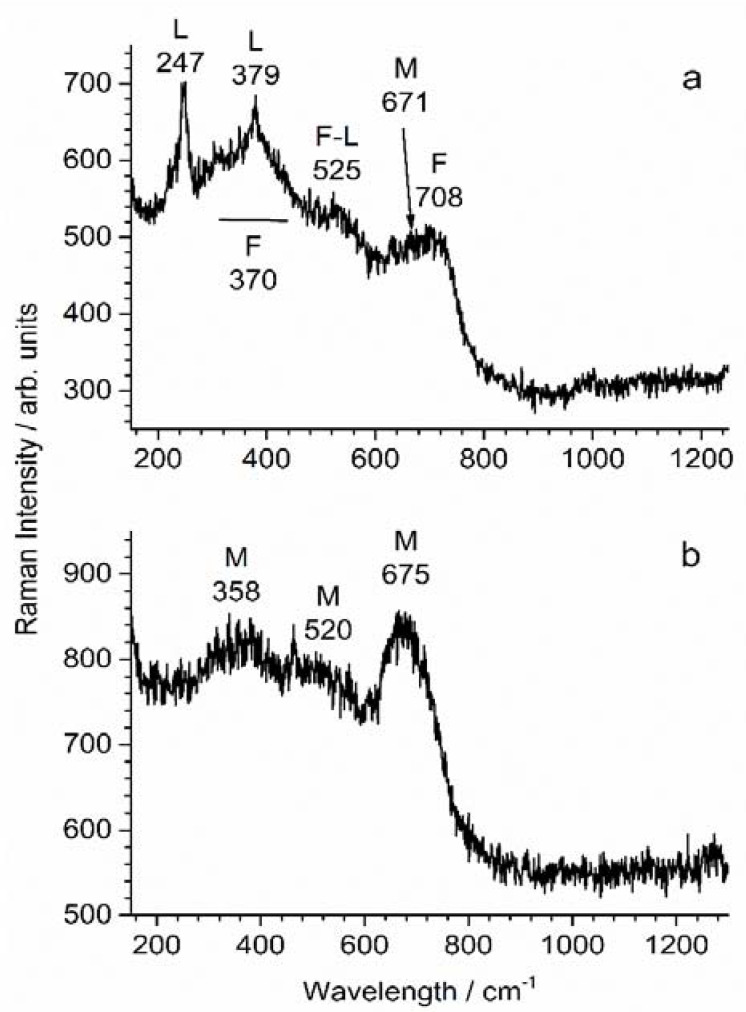
µ-Raman spectroscopy analysis of the surface of coupon WE_CP1_; G = goethite, M = magnetite/maghemite. See text for the interpretation of spectra (**a**,**b**).

The analysis of coupon WE_CP2_ gave similar results. The obtained Raman spectra (Figure 13) confirmed that corrosion products did form. Spectrum (a) revealed the presence of lepidocrocite γ-FeOOH, clearly identified by its two main peaks at 247 cm^−1^ and 379 cm^−1^ [43]. However, broad vibration bands are also visible at 708 cm^−1^, 370 cm^−1^ and 525 cm^−1^. They correspond to ferrihydrite, a poorly crystallized and poorly ordered hydrated Fe(III)-oxyhydroxide [47]. The main peak of magnetite is visible at 671 cm^−1^ as a shoulder of the main band of ferrihydrite. The presence of magnetite could be demonstrated more clearly via the analysis of other zones of the steel surface, as illustrated by spectrum (b).

It must be noted that CaCO_3_ phases could not be detected. First, it is possible that the conditions required for the formation of a calcareous deposit were not met. Secondly, µ-Raman spectroscopy is a local characterization technique, and if CaCO_3_ formed as a minor component, for instance, in the pores of the magnetite layer, it could have been missed. In any case, the decrease with time of the residual corrosion rate for coupon WE_CP1_ can be attributed to the formation of a thin layer of corrosion products.

In conclusion, for both WE_CP1_ and WE_CP2_ coupons, the steel surface proved to be covered by a layer mainly composed of magnetite and various Fe(III) oxyhydroxides. The predominance of magnetite, already observed in previous studies [20,27], can be attributed to the increase of the interfacial pH [48] induced by the cathodic reactions (see Equations (1) and (2)). Moreover, the cathodic polarization can also induce the reduction of Fe(III) to Fe(II) and thus the reduction of FeOOH compounds into magnetite. 

The EIS results, which showed a behavior typical of conductive porous electrode, can then clearly be attributed to the presence of the magnetite-containing layer. Magnetite is an electronic conductor with low resistivity [49], and consequently O_2_ molecules can be reduced on the walls of the pores of this layer. The term “magnetite layer” may not actually be appropriate. Magnetite was also identified in the pores of the soil at the very vicinity of the steel surface. The “porous electrode” behavior observed via EIS might not be due, strictly speaking, to a porous magnetite layer but rather to a small layer of soil where the pore walls could be covered with interconnected magnetite particles. This would explain how the small amount of magnetite resulting from the residual corrosion process could give rise to this porous electrode behavior.

The higher protective ability of the layer formed at −1.0 V vs. Cu/CuSO_4_ is not linked to its composition because the corrosion products identified for coupon WE_CP2_ polarized at −1.2 V vs. Cu/CuSO_4_ proved rather similar. It is therefore due to differences in terms of porosity, compactness and/or adhesion properties. The layer formed at −1.0 V vs. Cu/CuSO_4_ is more protective because it has fewer defects, which can be attributed to the fact that water reduction is not active. Actually, the release of H_2_ bubbles at −1.2 V vs. Cu/CuSO_4_ may have finally damaged this layer, which would explain the increase of the residual corrosion rate observed after day 63 (Figure 11). Water molecules are present in the pores of the corrosion product layer and at the surface of the steel. H_2_O reduction can then take place directly at the metal surface, and H_2_ molecules can then accumulate on this surface, finally forming H_2_ bubbles. If trapped between the metal and the protective layer, the bubbles would induce mechanical stresses and eventually cracks in the layer. The H_2_ bubbles would then be released in the soil through these cracks, and this H_2_ flow would moreover remove some magnetite particles from the metal surface, move them and finally set them on the pore walls of the soil.

## 4. Conclusions

The application of cathodic protection led, in the experimental conditions considered here, to the formation of a magnetite-rich layer, which resulted from the residual corrosion process occurring under CP [20,23,24,25,26,27]. For both applied potentials, EIS revealed that the steel surface behaved as a porous conductive electrode, which can be attributed to the presence of the magnetite-rich layer.

The voltammetry analysis revealed that steel dissolution was reduced from 330 µm yr^−1^ (after 7 days at OCP) to 7 µm yr^−1^ (after 70 days of CP) when the applied cathodic potential *E*_CP_ was −1.0 V vs. Cu/CuSO_4_. Consequently, the *E*_CP_ of −1.0 V vs. Cu/CuSO_4_ led to the full protection of steel as *τ*_rc_ was determined at a constant value smaller than 10 µm yr^−1^ (as recommended by NF EN 12954 standard [14]) toward the end of the experiment. The progressive decrease of *τ*_rc_ with time can be attributed to the growth of the layer of (residual) corrosion products. This layer can then be considered as protective at *E*_CP_ = −1.0 V vs. Cu/CuSO_4_. The modelling of EIS results proved consistent with voltammetry analysis and demonstrated that the porous conductive layer forming grew more and more protective with time. 

In contrast, voltammetry revealed that the polarization of steel at *E_C_*_P_ = −1.2 V vs. Cu/CuSO_4_ led to an average *τ*_rc_ value of 13 µm yr^−1^ after day 70, while it reached a smaller value of 8 µm yr^−1^ before. EIS results proved consistent and showed that the protective ability of this layer tended to decrease slightly after day 63. The variations of the residual corrosion rate are associated with an increase of soil electrolyte resistance *R*_s_ that may be due to the accumulation of H_2_ bubbles on the steel surface. The sporadic release of such bubbles would damage the protective layer and explain the higher residual corrosion rate.

## Figures and Tables

**Table 1:** Chemical composition of the electrolyte solution.

Compound	NaCl	CaCl_2_ 2H_2_O	MgCl 6H_2_O	NaSO_4_ 7H_2_O	NaHCO_3_
Conc. (g/L)	0.5844	0.2940	0.2033	0.4022	0.1680

**Table 2:** Fitted values of EIS data for both coupons at OCP (day 7).

Coupon	*R*_s_ (Ω cm^2^)	*n*	*R*_t_ (Ω cm^2^)	*R*_d_ (Ω cm^2^)	*t*_d_ (s)	*Q*_dl_ (F cm^−2^ s^n−1^)
WE_CP1_	1864	0.70	566	841	0.39	4.2 × 10^−4^
WE_CP2_	1816	0.72	455	1818	0.35	2.8 × 10^−4^

**Table 3:** Fitted values of EIS data for coupons WE_CP1_ and WE_CP2_ under CP.

Coupon	Time (d)	*R*_s_ (Ω cm^2^)	*R*_1_ (Ω cm^2^)	*n*	*Q*_1_ (F cm^−2^ s^n−1^)
WE_CP1_	42	940	659	0.60	2.8 × 10^−6^
	49	898	834	0.59	4.2 × 10^−6^
	63	838	1179	0.59	4.5 × 10^−6^
	70	834	1255	0.59	4.4 × 10^−6^
	77	930	1362	0.59	4.2 × 10^−6^
	84	916	1534	0.58	5.3 × 10^−6^
	90	926	1661	0.56	6.3 × 10^−6^
WE_CP2_	42	1711	595	0.53	7.5 × 10^−6^
	49	1852	707	0.52	6.4 × 10^−6^
	63	2142	1334	0.58	3.1 × 10^−6^
	70	2241	1318	0.57	3.6 × 10^−6^
	77	2382	1253	0.58	3.5 × 10^−6^
	84	2382	1254	0.59	2.8 × 10^−6^
	90	2425	1230	0.58	3.2 × 10^−6^

**Table 4:** Summary of fitted parameters computed from mathematical modelling for coupon WE_CP1_ (CSE = copper/copper sulfate electrode).

Day	*E*_corr_/ V_CSE_	*j*_corr_/ A cm^−2^	*j*_A_(*E*_CP_)/ A cm^−2^	*j*_lim_/ A cm^−2^	*b*_A/_ mV/dec	*b*_C_/ mV/dec	*τ*_corr_/ µm yr^−1^	*τ*_rc_/ µm yr^−1^
7 (OCP)	−0.85	2.86 × 10^−5^	-	−1.37 × 10^−4^	52	62	330	-
42	−0.54	2.71 × 10^−5^	1.62 × 10^−6^	−3.06 × 10^−4^	380	290	-	19
49	−0.45	1.52 × 10^−5^	1.64 × 10^−6^	−5.74 × 10^−4^	575	460	-	19
63	−0.37	3.01 × 10^−5^	1.59 × 10^−6^	−5.39 × 10^−4^	460	575	-	18
70	−0.45	2.45 × 10^−5^	1.92 × 10^−6^	−1.73 × 10^−4^	500	440	-	22
77	−0.41	2.10 × 10^−5^	6.23 × 10^−7^	−1.88 × 10^−4^	380	380	-	7
84	−0.38	2.45 × 10^−5^	6.07 × 10^−7^	−1.97 × 10^−4^	380	460	-	7
90	−0.36	2.78 × 10^−5^	6.20 × 10^−7^	−2.00 × 10^−4^	420	480	-	7

**Table 5:** Summary of the fitted parameters computed from mathematical modelling for coupon WE_CP2_ (CSE = copper/copper sulfate electrode). The values for *b*_C_ and *j*_lim_ are in brackets as the error is high (about ±50 %).

Day	*E*_corr_/ V_CSE_	*j*_corr_/ A cm^−2^	*j*_A_(*E*_CP_)/ A cm^−2^	*j*_lim_/ A cm^−2^	*b*_A_/ mV/dec	*b*_C_/ mV/dec	*τ*_corr_/ µm yr^−1^	*τ*_rc_/ µm yr^−1^
7 (OCP)	−0.85	3.15 × 10^−5^	-	−1.19 × 10^−4^	82	77	370	-
42	−0.64	1.95 × 10^−5^	8.96 × 10^−7^	(−2.3 × 10^−4^)	415	(190)	-	10
49	−0.55	3.09 × 10^−5^	1.32 × 10^−6^	(−5.0 × 10^−4^)	480	(560)	-	15
63	−0.45	2.71 × 10^−5^	7.24 × 10^−7^	(−4.7 × 10^−4^)	470	(720)	-	8
70	−0.39	3.36 × 10^−5^	7.94 × 10^−7^	(−7.3 ×10^−4^)	500	(960)	-	9
77	−0.41	3.14 × 10^−5^	1.29 × 10^−6^	(−5.9 × 10^−4^)	575	(410)	-	15
84	−0.43	2.87 × 10^−5^	1.10 × 10^−6^	(−5.6 × 10^−4^)	550	(885)	-	13
90	−0.38	2.57 × 10^−5^	1.00 × 10^−6^	(−4.3 × 10^−4^)	575	(885)	-	12

## Data Availability

The data generated during the current study are available from the corresponding author on reasonable request.
